# Pharmaceutical interventions in self-medication: A descriptive study in community pharmacies

**DOI:** 10.1016/j.rcsop.2026.100822

**Published:** 2026-07-09

**Authors:** Sabrina Bedhomme, Pierre Pradeleix, Elodie Martinet, Ines Ramos, Delphine Martineau, Elodie Lafarge, Bénédicte Pereton, Chantal Savanovitch

**Affiliations:** a Université Clermont Auvergne, Faculté de Pharmacie, F-63000 Clermont-Ferrand, France; b Université Clermont Auvergne, Research Unit ACCePPT, F-63000 Clermont-Ferrand, France; c Biostastistics and Data Management Unit, Department of Clinical Research and Innovation, CHU Clermont-Ferrand, F-63000 Clermont-Ferrand, France

**Keywords:** Self-care, Nonprescription drug, Community pharmacy, Drug related problem, Primary care

## Abstract

**Objective:**

The main objective of this study is to characterize self-medication Pharmaceutical Interventions (PI) carried out between 2017 and 2025.

**Methods:**

A descriptive study was conductive in 201 community pharmacies. Data were collected using GIPAMED notification grid.

**Results:**

This study looked at 3366 PIs involving selfmedication. The main reason for requesting drug by the patient or their representative, leading to an PI, was pain (in nearly one-third of cases). The molecules that caused the highest number of PIs were ibuprofen, paracetamol, oxomemazine, pheniramine in combination, and fosfomycin. The main reasons for PIs were “non-compliance with regulations: request for a prescription drug only”, “contraindication with the pathophysiological condition,” “non-indication with regard to the symptoms,” and “need for a medical consultation: limits of pharmacy advice.” The dispenser referred the patient for a consultation in 38.1% of cases. The solution proposed by the pharmacist was accepted by the interlocutor in more than 90% of cases.

**Conclusions:**

The overall characterization of PIs provided a descriptive basis for identifying the most common types of PIs, the molecules involved, and how they are resolved. This data highlights areas requiring vigilance when dispensing nonprescription drugs. The prospective PI database developed has thus made a significant contribution to primary care research and, more specifically, to community pharmacy research.

## 1 Introduction

Self-medication (SM) is becoming increasingly common in France.^1^ In 2024, 74% of the French population reported purchasing nonprescription health products from community pharmacies, with particularly high use among young adults.2., 3.

Although the self-medication sector remains less developed in France than in several other European countries, it represents a valuable opportunity to improve healthcare delivery and reduce the burden on medical practices, which are currently under considerable pressure. Indeed, the ongoing shortage of healthcare professionals is limiting access to care for many patients. Responsible self-medication may therefore contribute to reducing regional disparities in healthcare access.

However, there is currently no universally accepted definition of self-medication, and relatively few studies have investigated this practice.^4^ According to the World Health Organization (WHO), responsible self-medication consists of “treating illnesses with authorized over-the-counter (OTC) medicines that are safe and effective under the conditions of use indicated”.^5^ In its most recent dictionary edition (2001), the French National Academy of Pharmacy defines self-medication as the use by a patient of one or more OTC medicines dispensed by a pharmacist without a physician's prescription. This definition refers specifically to nonprescription medicines and therefore excludes the use of medications already present in the household medicine cabinet, including those previously prescribed by a physician and subsequently reused at the patient's own initiative. The present study was based on this definition.^6^

It should be noted that inappropriate self-medication may lead to several risks, including misuse, overdose, dependence, delayed diagnosis or treatment, adverse drug reactions, and drug–drug interactions.7., 8. Furthermore, drug-related problems have been shown to generate healthcare costs that are equal to, or even greater than, the cost of the medicines themselves.^9^

The primary role of the community pharmacist is to ensure the safe and appropriate dispensing of medicines, particularly when no prescription is presented. In the context of self-medication, when a patient makes a spontaneous request for a medicine, the pharmacist conducts a structured interview based on the A.C.R.O.P.O.L.E. method, developed by the French National Council of the Order of Pharmacists to optimize the management of patients presenting to a pharmacy without a prescription.^10^

The pharmacist is responsible for identifying and preventing potential drug-related problems through patient interviews and, when available, by consulting the patient's medication history or pharmaceutical record, which lists medicines dispensed during the previous 12 months.11., 12. When necessary, the pharmacist may perform a pharmaceutical intervention (PI), defined by the French Society of Clinical Pharmacy as “a proposal to modify therapy initiated by the pharmacist, concerning one or more health products, and involving the identification, prevention, and resolution of therapy-related problems in a given patient”.^13^

The main objective of this study was to characterize pharmaceutical interventions related to self-medication performed in the Auvergne region of France between 2017 and 2025.

## 2 Materials and methods

### 2.1 Study design

This descriptive cross-sectional study investigated pharmaceutical interventions (PIs) related to self-medication.

The PIs were collected in 201 community pharmacies, which volunteered to participate in the study while hosting a pharmacy student during their professional internship under the supervision of a pharmacist holding an official internship preceptorship accreditation.

### 2.2 Data collection

The ACCePPT research team developed GIPAMED (Grid for Pharmaceutical Self-Medication Interventions), a standardized data collection tool specifically designed to record self-medication-related pharmaceutical interventions in community pharmacies.^14^ The intern validity of the grid was assessed by studying inter-item correlations and multidimensional analyses to ensure that the items grouped together in the different dimensions studied. The accuracy of the instrument was assessed according to inter-judge reproducibility, the ability to produce comparable results when the measurement is repeated and evaluated by two different pharmacists while the individual's condition remains stable. The kappa concordance coefficients and intra-class correlation coefficients were calculated. The validity of the external structure was based on the study of the concordance between community pharmacists and expert opinions used as a decision-making reference framework.^14^

Pharmaceutical interventions were reported and documented using the GIPAMED grid. Data collection was conducted during the first week of each month, from February through May, between 2017 and 2025. These data collection periods, included in the students' internship periods, were chosen in order to keep the data collection systematic during a short period. Data collectors were students under the supervision of internship masters in the pharmacy. A meeting was held beforehand to present the study and train both of them in the use of the reporting grid.

The study included spontaneous patient requests for medicines in the context of self-medication that resulted in a pharmaceutical intervention.

The criteria of exclusion were: requests related to a prescription, requests subject to advice, requests concerning health products other than medicines (e.g., dietary supplements), veterinary medicines, medicines obtained abroad, and medicines withdrawn from the French market.

According to the GIPAMED grid, the following information was collected for each pharmaceutical intervention: the requested medicine (brand name, type, dosage, and pharmaceutical form), patient sex and age, reason for the intervention (regulatory non-compliance, limitations of pharmacy advice, contraindications, not indicated medicine in view of the symptomatology reported by the patient, adherence issues, risk of overdose, or drug interactions), method used to detect the intervention (patient interview, pharmaceutical record, or medication history), action proposed by the pharmacist (dose adjustment, therapeutic alternative, referral for medical consultation, or health and lifestyle advice), outcome of the intervention (accepted or refused by the patient or patient representative), and, when applicable, the reason for refusal. The grid also included a free-text field allowing detailed description of each intervention.

Free-text fields were subjected to double independent review and exploratorily analyzed through a recoding process to verify the consistency of the coded data; duplicate entries were identified and removed at this stage.

Additional variables were recorded for the present study, including the pharmacotherapeutic (PT) class and Anatomical Therapeutic Chemical (ATC) classification of the requested medicine, the relevant medical specialty, and the reason for the request (i.e., the presenting complaint reported by the patient). Municipalities were categorized using the seven-level population density classification developed by the French National Institute of Statistics and Economic Studies (INSEE).^15^

### 2.3 Statistical analysis

Pharmaceutical interventions were recorded online through a secure platform hosted on the RENATER network, the French national telecommunications network for education and research. Data were subsequently exported to Microsoft Excel® (Office 2021; Microsoft Corporation, Redmond, WA, USA).

Each pharmaceutical intervention was reviewed individually to verify data completeness and consistency. Statistical analyses were performed using Stata software (version 13; StataCorp, College Station, TX, USA).

Categorical variables were described using frequencies and percentages. Descriptive and comparative analyses were performed, with statistical significance defined as a two-sided *p*-value ≤0.05.

### 2.4 Ethical and regulatory considerations

The study protocol was submitted to the South-East VI Human Research Ethics Committee, which issued an ethics approval certificate (Ref. 2016/17 CE) and waived the requirement for informed consent.

The study did not fall within the scope of research involving human participants (RIPH) as defined by the French Jarde Law and its implementing regulations. No patients were directly interviewed by the investigators, as the study focused on pharmacists' professional practice and relied exclusively on data reported during routine pharmaceutical care activities.

The study was conducted in accordance with the Declaration of Helsinki. With regard to personal data protection, the study was registered with the local representative of the French Data Protection Authority (CNIL) at Université Clermont Auvergne (Ref. 2016–13).

## 3 Results

A total of 3366 pharmaceutical interventions (PIs) recorded between 2017 and 2025 were included in the analysis Population characteristics.

The characteristics of the study population are presented in Table I.

**Table I:** Characteristics of the study population concerned by PIs (n,% and CI95%).

Characteristics	n	%	CI95%
**Sex**			
Women	1765	52.4%	[50,7% - 54,1%]
Men	1598	47.5%	[45,8% - 49,2%]
No response	3	0.1%	
**Age groups**			
<15 years	219	6.5%	[5,7% - 7,3%]
15–44 years	1379	41.0%	[39,3% - 42,6%]
45–64 years	879	26.1%	[24,6% - 27,6%]
≥ 65 years	602	17.9%	[16,6% - 19,2%]
No response	287	8.5%	
**Interlocutor**			
Patient	2612	77.6%	[76,2% - 79,0%]
proxy	745	22.1%	[20,7% - 23,5%]
No response	9	0.3%	
**Pregnant women**	100	3%	[2,4% - 3,5%]
**Breastfeeding women**	8	0.2%	[0,1% - 0,4%]
**Population density**			
Hight density (NC1)	1530	45.5%	[43,8% - 47,1%]
Intermediate (NC 2–4)	1185	35.2%	[33,6% - 36,8%]
Rural (NC 5–7)	609	18.1%	[16,8% - 19,4%]
No response	42	1.2%	

The male-to-female ratio was 0.91. The mean age of patients involved in a PI was 44 years (range: 2–95 years). Most PIs were performed in municipalities with high or intermediate population density and to a lesser extent in rural areas (Table I). The population affected by PIs is significantly older in rural areas than in other areas: 24.5% of PIs were generated among those aged 65 and over in rural areas, compared with 14.9% among those aged 15–44 and 20.2% among those aged 45–64 (*p* = 0.001).

### 3.1 Methods used to detect pharmaceutical intervention

With regard to methods of detecting PIs, dialogue was used most often (95.2% of PIs, *n* = 3203). The patient's medication history and/or the pharmaceutical record remain very rare, accounting for 12.7% (*n* = 429) and 3.2% of PIs (*n* = 109) respectively. Patient's interview was by far the most frequently used method for detecting PIs, accounting for 95.2% of interventions (n = 3203).

Consultation of the patient's medication history and pharmaceutical record was considerably less frequent, being involved in 12.7% (n = 429) and 3.2% (n = 109) of PIs, respectively.

### 3.2 Main medical fields and presenting complaints

**Fig. 1:**
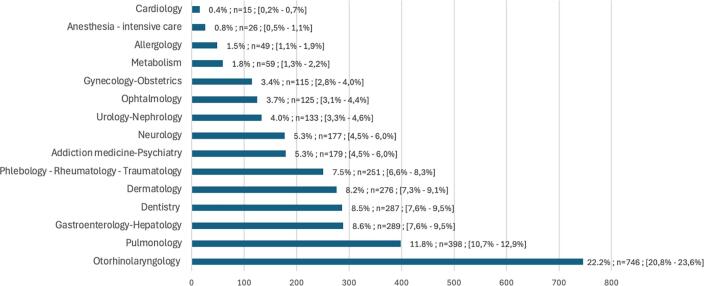
Distribution of main medical fields by requests that led to a PI (*n* ≥ 5 PIs). No response = 236. Data labels indicate the percentage, the corresponding number of interventions (n) and CI 95%.

Fig. 1 presents the main medical fields.

Pain was the most common presenting complaint associated with spontaneous requests leading to a PI (*n* = 1003; 29.8%).

Pain-related requests were mainly associated with the fields of phlebology-rheumatology-traumatology (*n* = 217; 21.6%), dentistry (*n* = 215; 21.4%), and neurology (*n* = 172; 17.1%).

Other frequent presenting complaints (≥ 3.0% of PIs) included colds (*n* = 440; 13.1%), cough.

(*n* = 364; 10.8%), suspected cystitis (*n* = 117; 3.5%), and insomnia (*n* = 108; 3.2%).

The presenting complaint was not documented in 4.9% of PIs (*n* = 166).

### 3.3 Main molecules associated with pharmaceutical intervention

The molecules most frequently associated with PIs were ibuprofen (*n* = 374; 11.1%), paracetamol (*n* = 211; 6.3%), oxomemazine (*n* = 162; 4.8%), pheniramine-containing combinations (*n* = 98; 2.9%), fosfomycin (*n* = 97; 2.9%), desloratadine (*n* = 71; 2.1%), pseudoephedrine-containing combinations (*n* = 61, 1.8%), doxylamine (*n* = 59; 1.8%), acetylsalicylic acid (*n* = 50; 1.5%), and paracetamol-codeine combinations (*n* = 43; 1.3%).

### 3.4 Main drug classes and associated molecules

Table II presents the main ATC level 1 and 2 drug classes according to the medical field of the request and the patients' main complaints. The most frequently represented ATC classes were:

**Table II:** Distribution of the main ATC level 1 and 2 drug classes according to the medical field of the request and the patients' main complaints. Data are presented as percentage and frequencies (n, %).

Main ATC level 1 classes	Main ATC level 2 classes	Main medical areas of the request	Main patient's complaints
(%, number)	(%, number)	(number)	(number)
Respiratory system (34,1% n = 1148)	Cough and cold preparation (34,6%, *n* = 383)	Otorhinolaryngology (*n* = 153)	Cold (*n* = 119) Flu-like symptoms (*n* = 16)
		Pulmonology (*n* = 110)	Cough (n = 110)
		Pulmonology (n = 47)	Cough (*n* = 47)
	Antihistamine for systemic use (31,8%, n = 352)	Pulmonology (*n* = 135)	Cough (*n* = 134)
		Addictologie-psychiatrie (n = 15)	Insomnia (n = 15)
		Otorhinolaryngology (*n* = 52)	Allergic rhinitis (*n* = 48)
		Allergology (*n* = 43)	Allergic reaction (n = 30)
		Dermatology (n = 13)	Pruritus (n = 4); Chickenpox (n = 4)
		Addictologie-Psychiatrie (*n* = 57)	Insomnia (*n* = 57)
	Nasal preparations (19,1%, *n* = 212)	Otorhinolaryngology (*n* = 181)	Rhume (*n* = 163) Allergic rhinitis (n = 13)
Musculo-skeletal system (16,80%, n = 567)	Antiinflammatory and antirheumatic products (84,4%, n = 476)	Dentistry (*n* = 197)	Pain (*n* = 357)
		Neurology (n = 80)	
		Phlebology-rheumatology-traumatology (n = 51)	
		Otorhinolaryngology (n = 32)	
	Topical products for joint and muscular pain (13,7%, *n* = 77)	Phlebology-rheumatology-traumatology (*n* = 67)	Pain (*n* = 63)
Nervous system (15,4%, n = 518	Analgesics (75%, n = 387)	Neurology (n = 57) Otorhinolaryngology (*n* = 46) Phlebology-rheumatology-traumatology (*n* = 39)	Pain (*n* = 202)
		Neurology (n = 25) Phlebology-rheumatology-traumatology (n = 23) Dentistry (n = 16)	Pain (*n* = −78)
	Psycholeptics (11,8%, n = 61)	Addictology-psychiatry (n = 26)	Insomnia (n = 20)
		Addictology-psychiatry (*n* = 22)	Anxiety (n = 13) Insomnia (n = 5)
Alimentary tract and metabolism (10,60%, n = 358)	Antidiarrheals, intestinal antiinflammatory/antiinfective agents (28,9%, *n* = 91)	Gastroenterology-hepatology (n = 77)	Diarrhoea (*n* = 58)
	Drugs for constipation (19,7%, n = 62)	Gastroenterology-hepatology (n = 59)	Constipation (n = 52)
	Drugs for acide related disorders (19,7%, n = 62)	Gastroenterology-hepatology (n = 42)	Gastroesophageal reflux disease (n = 20)
	Stomatological preparations (11,4%, n = 36)	Dentistry (n = 29)	Prophylaxis (n = 13) Other buccal illness (n = 11)
Dermatologicals (7,50%, n = 251)	Antiseptics and disinfectants (26,2%, n = 64)	Dermatology (n = 49)	Wound (n = 26) Other dermatosis (n = 15)
	Corticosteroid, dermatological preparations (23,0%, n = 56)	Dermatology (n = 44)	Eczema (n = 27) Pruritus (n = 7)
	Antibiotics et chemotherapeutics for dermatological use (15,2%, n = 37)	Dermatology (n = 22)	Other dermatosis (n = 12) Wound (n = 7)
	Antifungals for dermatological use (13,9%, *n* = 34)	Dermatology (n = 30)	Mycosis (n = 15) Other dermatosis (n = 9)
Antiinfectives for systemic use (4,8%, n = 161)	Antibacterial for systemic use (90,6%, n = 144)	Urology-nephrology (n = 109)	Cystitis (n = 108)
Sensory organs (4,8%, n = 161)	Ophtalmologicals (71,9%, n = 115)	Ophtalmology (n = 27)	Affection palpébrale (n = 13) Conjonctivite (n = 11)
		Ophtalmology (n = 22)	Conjonctivite (n = 15) Affection palpébrale (n = 4)
		Ophtalmology (n = 22)	Conjonctivite (n = 10) Plaie (n = 8)
	Otologicals (28,1%, n = 45)	Otorhinolaryngology (n = 27)	Pain (*n* = 27)
		Otorhinolaryngology (n = 17)	Pain (n = 9) Bouchon de cérumen (n = 6)
Genitourinary system and sex hormones	Sex hormones and modulators of the genital system	Gynecology-obstetrics	Emergency contraception (n = 24)
(2,6%, n = 88)	(54,7%, n = 47)	(n = 38)	Contraception (n = 10)
	Gynécological antiinfective and antiseptics (26,7%, n = 23)	Gynecology-obstetrics (n = 16)	Vaginal condiction (n = 14)
		Urology-nephrology (n = 7)	Cystitis (n = 7)
	Urologicals (18,6%, n = 16)	Urology-nephrology (n = 14)	Erectil dysfonction (n = 13)

#### 3.4.1 Respiratory system

The respiratory system class accounted for 34.1% of all PIs (*n* = 1148). The most frequently involved medicines were cough and cold preparations (*n* = 383) and systemic antihistamines (n= 352).

Among antihistamines, oxomemazine was particularly prominent (n = 162), mainly associated with cough-related requests, but also with insomnia-related requests (*n* = 15).

#### 3.4.2 Musculoskeletal system

The musculoskeletal system class represented 16.8% of PIs (*n* = 567) and consisted mainly of anti-inflammatory and antirheumatic drugs (*n* = 476), which were predominantly associated with pain-related complaints.

#### 3.4.3 Nervous system

The nervous system class accounted for 15.4% of PIs (*n* = 518), including analgesics (*n* = 387), which were also predominantly associated with pain.

#### 3.4.4 Alimentary tract and metabolism

This class represented 10.6% of PIs (*n* = 358) and was mainly composed of antidiarrheal agents (*n* = 91) and medicines used to treat constipation (*n* = 62).

#### 3.4.5 Dermatologicals

Dermatologicals accounted for 7.5% of PIs (*n* = 251), including antiseptics and disinfectants (*n* = 64) as well as topical corticosteroids.

#### 3.4.6 Anti-infectives for systemic use

This class represented 4.8% of PIs (*n* = 161) and was largely dominated by fosfomycin (*n* = 108), which accounted for approximately 67% of interventions within this category.

#### 3.4.7 Sensory organs

Medicines classified under Sensory Organs class represented 4.8% of PIs (n = 161), including ophthalmologicals (*n* = 115) and otologicals (*n* = 45).

#### 3.4.8 Genitourinary system and sex hormones

This class accounted for 2.6% of PIs (*n* = 88) and mainly involved requests for emergency contraception (*n* = 24) and anti-infectives or antiseptics intended for gynecological use (*n* = 23).

### 3.5 Main reasons for pharmaceutical interventions

**Fig. 2:**
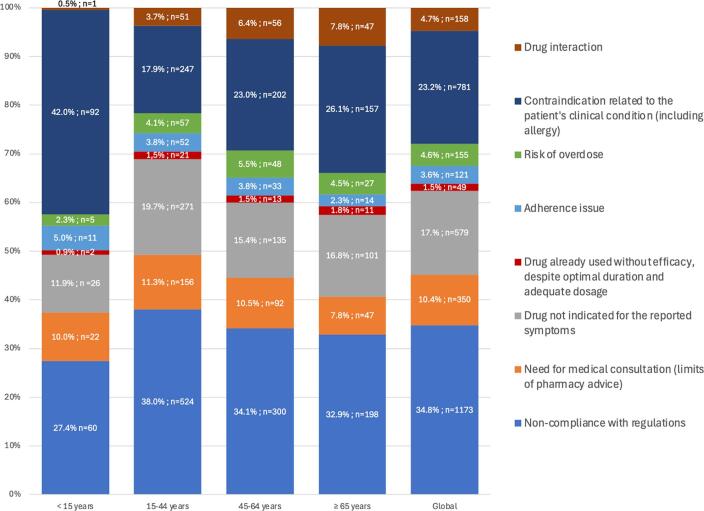
Distribution of pharmaceutical interventions related to self-medication by intervention reason across age groups and globally. Each stacked bar represents 100% of pharmaceutical interventions within an age group and globally. Data labels indicate the percentage and corresponding number of interventions (*n*).

The reasons for pharmaceutical interventions are presented below in descending order of frequency (Fig. 2).

#### 3.5.1 Request for a prescription-only medicine (non-compliance with regulation)

This was the most frequent reason for intervention, accounting for 34.8% of all PIs (*n* = 1173).

The medical fields most commonly associated with these interventions were otorhinolaryngology (*n* = 203; 18.7%), urology-nephrology (*n* = 123; 11.5%), dermatology (n = 123; 11.3%), and pulmonology (n = 123; 11.3%).

The most frequent presenting complaints were pain (*n* = 257; 23.7%), suspected cystitis (n = 108; 9.9%), and cough (*n* = 101; 9.3%).

The ATC classes most commonly involved were systemic antibacterials (*n* = 142; 12.2%), cough and cold preparations (*n* = 127; 11.7%), systemic antihistamines (*n* = 99; 8.5%), and analgesics (*n* = 96; 8.2%).

The molecules most frequently involved were fosfomycin (*n* = 107; 9.1%) and desloratadine (*n* = 82; 7.0%).

#### 3.5.2 Contraindication related to the patient's clinical Condition (Including allergy)

This reason accounted for 23.2% of interventions (*n* = 781).

The associated fields were primarily otorhinolaryngology (*n* = 274; 37.4%), dentistry (*n* = 113; 15.4%), and pulmonology (*n* = 93; 12.7%).

The most common presenting complaints were pain (*n* = 265; 37.1%) and colds (*n* = 210; 27.8%).

#### 3.5.3 Medication not indicated for the reported symptoms

A total of 579 interventions (17.2%) involved a medicine that was not indicated for the patient's reported symptoms.

The main fields involved were pulmonology (*n* = 145; 25.3%), otorhinolaryngology (*n* = 126; 22.0%), and gastroenterology-hepatology (n = 99; 17.3%).

The most common presenting complaints were cough (*n* = 144; 25.5%) and pain (*n* = 90; 17.5%).

#### 3.5.4 Need for medical consultation (limits of pharmacy advice)

This reason accounted for 10.4% of interventions (*n* = 350).

The main presenting complaints were pain (n = 142; 42.6%) and wounds (*n* = 42; 12.6%).

The associated fields were dentistry (*n* = 87; 25.0%), dermatology (*n* = 81; 23.3%), otorhinolaryngology (*n* = 49; 14.1%), and ophthalmology (*n* = 45; 12.9%).

#### 3.5.5 Drug interaction

A total of 158 interventions (4.7%) involved a potential drug interaction.

The molecules most frequently involved were ibuprofen (*n* = 50), St. John's wort (*n* = 24), and acetylsalicylic acid (*n* = 16).

Ibuprofen and acetylsalicylic acid mainly interacted with anticoagulants (fluindione, apixaban, rivaroxaban, and warfarin), whereas St. John's wort primarily interacted with estrogen-progestogen contraceptives.

#### 3.5.6 Risk of overdose

A total of 155 interventions (4.6%) involved a risk of overdose.

These interventions concerned requests for medicines already being used either through self-medication (*n* = 101) or as part of an ongoing prescribed treatment (*n* = 54).

The most frequent situation involved a risk of paracetamol overdose, whether paracetamol was requested alone or as part of a combination product (*n* = 112).

#### 3.5.7 Adherence issues

A total of 121 interventions (3.6%) were related to adherence issues. These involved supratherapeutic dosing in 58.8% of cases (*n* = 67); subtherapeutic dosing in 37.7% of cases (*n* = 43) and appropriate dosing but insufficient treatment duration in 3.5% of cases (n = 4).

These situations mainly involved analgesics, particularly paracetamol (*n* = 60) and nonsteroidal anti-inflammatory drugs (NSAIDs) (*n* = 17).

#### 3.5.8 Drug already used without efficacy despite optimal duration and adequate dosage

This reason accounted for 1.6% of interventions (n = 49).

The medicines most frequently involved were cough and cold preparations (n = 10) and analgesics (*n* = 9).

The distribution of pharmaceutical intervention by intervention reason across age groups is presented in Fig. 2. The age-related contraindication is a major reason for those under 15, while the risk of drug interactions tends to increase with age.

### 3.6 Actions proposed by the pharmacist

At the conclusion of the pharmaceutical intervention, pharmacists could propose one or more actions to the patient or patient representative.

A therapeutic alternative to the requested medicine was proposed in 92.6% of cases (*n* = 2117). In most situations, this alternative consisted of a nonprescription medicine (*n* = 1561), although dietary supplements (*n* = 297) and ingestible medical devices (*n* = 259) were also recommended.

Referral for medical consultation was proposed in 38.1% of cases (*n* = 1282).

Health and lifestyle advice was provided in 23.9% of cases (*n* = 803), while dosage adjustment or modification of the administration schedule was recommended in 8.7% of cases (*n* = 293).

### 3.7 Acceptance of pharmacists' recommendations

The actions proposed by pharmacists were accepted in 91.4% of cases (*n* = 3076).

In half of the cases in which the recommendation was refused, the reason was that the patient was accustomed to using the requested medicine or specifically wished to obtain that medicine (*n* = 149; 51.4%).

The complete distribution of refusal reasons is presented in Table III.

**Table III:** Reasons for rejecting the action proposed by the pharmacist (n, %).

Reason for refusal	n	%
Medication use habit	80	27.6%
Only wants the requested product	69	23.8%
Alternative already used without effectiveness	37	12.8%
Medical consultation	37	12.8%
Consultation refusal	18	6.2%
Representative or decision subject to the opinion of a relative	16	5.5%
Inefficient alternative	13	4.5%
Too expensive alternative	7	2.4%
Alternative already in his possession	6	2.1%
Refusal of alternative and prescription of the requested medication	5	1.7%
No response	2	0.7%
**Total**	290	

## 4 Discussion

In summary, 3366 pharmaceutical interventions related to self-medication were analyzed over a nine-year period. Pain was the most common presenting complaint associated with spontaneous requests leading to a pharmaceutical intervention, accounting for nearly one-third of all cases. The molecules most frequently involved were ibuprofen, paracetamol, oxomemazine, pheniramine-containing combinations, and fosfomycin. The principal reasons for intervention were requests for prescription-only medicines, contraindications related to the patient's clinical condition, use of medicines not indicated for the reported symptoms, and situations requiring referral for medical consultation because they exceeded the scope of pharmacy advice. In more than one-third of cases (31.8%), patients were referred for medical consultation, and pharmacists' recommendations were accepted in over 90% of interventions.

The nature of Drug Related Problems associated with prescribed medicines and their impact on patients' well-being are well described, as in the 2023 Danish study by Kaae S. et al.^16^ Some of their findings are reflected in our study, such as the misuse of paracetamol due to a lack of information, which can lead to an overdose. Pain was the leading presenting complaint associated with requests for self-medication in our study. This finding is consistent with national data showing that a substantial proportion of the French population experiences significant physical pain, including young adults, particularly in the form of headaches and low back pain.^17^ Consequently, self-medication is frequently used to manage these symptoms. However, the French National Agency for Medicines and Health Products Safety recommends that nonprescription medicines should be used only for common, mild conditions and for limited periods, generally not exceeding three to five days for pain management.^18^

The significant proportion of pain-related PIs in our study highlights the important role of pharmacists in managing patients with painful symptoms. This is in in accordance with the literature that show they acting whether by ensuring the proper use of drugs, referring them to another healthcare professional if necessary, or identifying and preventing the misuse of painkillers. These results align with existing literature documenting pharmacist interventions aimed at promoting appropriate drug use, facilitating referral to other healthcare professionals when indicated, and preventing analgesic misuse.^18^ Their scope of action extends from independent practice to collaborative engagement within multidisciplinary teams.^19^ In France, until 2017, codeine, a level II analgesic, was available without prescription,^20^ allowing pharmacists to recommend it as a second-line treatment when non-opioid analgesics proved insufficient.^21^ More recently, the French National Authority for Health formally recognized the role of community pharmacists in the management of chronic pain, including medicine dispensing, identification of misuse, and therapeutic education.^22^ This role was further strengthened in 2024 through the implementation of pharmaceutical interviews dedicated to patients receiving step II opioid therapy.^23^

Optimizing analgesic self-medication support and pain-related referral pathways within the healthcare system remain areas for development, subject to adequate availability and targeted training of pharmacy staff.^24^, 25.

The development of collaborative care protocols involving pharmacists and physicians, similar to those already implemented for conditions such as cystitis and tonsillitis, may represent a valuable opportunity to improve patient care and access to treatment.^26^

Among the medicines most frequently associated with pharmaceutical interventions, ibuprofen and paracetamol were predominant. These two molecules are included in Finland's national classification of high-risk medicines. Among them are seven over-the-counter medicines, including ibuprofen, acetylsalicylic acid and paracetamol, which appear on this list because they can cause serious adverse effects.^27^

Other drugs that generated a high number of PIs included oxomemazine, pheniramine in combination, fosfomycin, desloratadine, and pseudoephedrine in combination.

Oxomemazine was primarily associated with interventions related to the request of medicines lacking an appropriate therapeutic indication, regardless of patient age. This medicine, commonly available as a cough syrup indicated for dry and irritating cough, was frequently requested by patients presenting with productive cough. Not only is oxomemazine not indicated in this context, but productive cough constitutes an important physiological defense mechanism of the respiratory tract and should generally not be suppressed. Furthermore, recent studies have reported a low level of evidence supporting the efficacy of H1 antihistamines, including oxomemazine, in the management of non-allergic dry cough.28., 29. Another example observed in our study involved requests for oxomemazine as a treatment for insomnia despite the absence of an approved indication for this use. Guerlays *et al.* Have described this misuse previously.^30^

Pheniramine-containing combinations and doxylamine were particularly associated with pharmaceutical interventions among patients aged 65 years and older. The principal reason for intervention was the presence of contraindications related to the patient's clinical condition, as first-generation H1 antihistamines possess significant anticholinergic properties. Most interventions concerned the risk of urinary retention in patients with uretroprostatic disorders, a population for whom these medicines are generally not recommended.^31^ Pharmaceutical intervention is particularly important in this case, given that anti-H1 drugs also significantly increase the risk of falls in elderly patients due to their sedative properties.^32^

For fosfomycin, the sole reason for intervention was regulatory non-compliance, as the medicine remained prescription-only throughout most of the study period. Fosfomycin is frequently requested in community pharmacies by women presenting with symptoms suggestive of uncomplicated cystitis, for which it is recommended as first-line therapy. The number of interventions related to fosfomycin is likely to decrease in the coming years following the publication of Decree No. 2024–550 of June 17, 2024, which authorizes the dispensing without prescription of certain antibiotics recommended for uncomplicated acute cystitis after completion of a rapid diagnostic orientation test.^33^ The high frequency of fosfomycin-related interventions observed in our study suggests a substantial unmet patient need in this area. Evaluations of similar pharmacist-led management protocols implemented in other countries, particularly Canada, have demonstrated their effectiveness and safety, as well as high levels of patient satisfaction regarding accessibility and quality of care.^34^, 35. Nevertheless, the long-term success of such protocols depends on strict adherence to referral criteria and recognition of warning signs requiring medical evaluation.

Desloratadine was also frequently involved in pharmaceutical interventions. Although several antihistamines are available without prescription, desloratadine remains highly requested because it is widely prescribed in France for allergic rhinitis and is due to a low risk of sedation.^36^ Since February 2025, the French National Agency for Medicines and Health Products Safety has authorized the sale of seven-tablet packs of desloratadine without prescription. As a result, interventions related to this medicine may become less frequent in the future.^37^

Pseudoephedrine-containing combinations were particularly associated with contraindication-related interventions, especially among patients aged 45–64 years. These medicines were frequently requested by individuals with uncontrolled high blood pressure despite known cardiovascular risks. In addition, pseudoephedrine has been associated with rare but potentially severe adverse events, including posterior reversible encephalopathy syndrome and reversible cerebral vasoconstriction syndrome. Consequently, health authorities issued recommendations in 2023 and 2024 discouraging its use for the treatment of common cold symptoms, ultimately leading to prescription-only status in France from December 2024 onward.38., 39.

Several limitations should be considered when interpreting the results of this study. First, the distribution of participating pharmacies was not balanced across rural, intermediate, and densely populated areas, which may limit the representativeness of the findings. This imbalance may reflect a selection bias inherent to the internship allocation process, as students tend to favor urban and semi-urban pharmacy settings over rural ones. Second, a seasonal bias may have been introduced because data collection was conducted exclusively between February and May each year. Consequently, the study mainly reflects winter and spring self-medication practices and may overrepresent medicines used for respiratory conditions such as allergies, colds, and cough.

However, there are few studies at both the national and international levels that systematically and structurally document PIs related to SM. The development of the standardized GIPAMED reporting tool enabled the creation of a prospective database specifically dedicated to self-medication-related interventions and allowed multidimensional analysis of situations, involving self-medication that present a potential risk to patients. The continuous collection of data over nine consecutive years also provides a unique opportunity to examine trends in self-medication practices and associated risks in relation to regulatory changes and evolving recommendations from health authorities.

The results of the study reflect the profiles of nonprescription drugs s that may cause iatrogenic effects in patients. Overall, our findings are consistent with the limited literature currently available on this topic. The principal reasons for self-medication requests involve pain and respiratory symptoms.^40^ While NSAIDs, analgesics, and medicines used to treat respiratory conditions account for a substantial proportion of pharmaceutical interventions.^41^ The role of pharmacists in preventing adverse drug reactions through pharmaceutical interventions is consistent with the findings of a 2026 Finnish study, which demonstrated that pharmacists contribute to patient safety by assessing self-medication needs and potential drug interactions, substituting inappropriate medications with suitable alternatives, and referring patients to a physician when clinically indicated.^42^

## 5 Conclusion

In conclusion, this study provides a comprehensive characterization of pharmaceutical interventions related to self-medication in community pharmacies. The analysis identified the most frequent types of interventions, the medicines involved, and the strategies used by pharmacists to resolve drug-related issues.

The analysis of PIs highlights medicines associated with the risk of iatrogenic adverse drug reactions and misuse, as well as points to watch out for depending on the patients' profiles.

These findings underscore the central role of community pharmacists as essential first-line healthcare professionals in ensuring medication safety within the self-care context. Greater institutional recognition of pharmaceutical interventions remains an important challenge. Their integration as a quality indicator of community pharmacy practice could further strengthen their contribution to patient care and support the continued evolution of the profession. Indeed, Mäkinen et al. introduces nationwide actions that could be utilised in other countries to enhance community pharmacies' involvement.^43^

## CRediT authorship contribution statement

**Sabrina Bedhomme:** Writing – original draft, Visualization, Validation, Supervision, Investigation, Funding acquisition, Conceptualization. **Pierre Pradeleix:** Writing – original draft, Visualization, Validation. **Elodie Martinet:** Writing – review & editing, Investigation, Data curation. **Ines Ramos:** Methodology, Formal analysis. **Delphine Martineau:** Writing – review & editing, Formal analysis. **Elodie Lafarge:** Investigation, Data curation. **Bénédicte Pereton:** Writing – review & editing, Validation, Investigation, Data curation. **Chantal Savanovitch:** Writing – original draft, Supervision, Methodology, Funding acquisition, Formal analysis, Conceptualization.

## Declaration of competing interest

The authors declare that they have no known competing financial interests or personal relationships that could have appeared to influence the work reported in this paper.
